# Renal Medullary Carcinoma in an Adolescent With Unknown Sickle Cell Trait

**DOI:** 10.7759/cureus.14473

**Published:** 2021-04-13

**Authors:** Brian D Noreña-Rengifo, Jorge Ochoa-Gaviria, Alejandro Vélez-Escobar, Juan P Muñoz, Marcela Riveros-Ángel

**Affiliations:** 1 Radiology, Universidad de Antioquia, Medellín, COL; 2 Radiology, Hospital Infantil San Vicente Fundación, Medellín, COL; 3 Radiology, Hospital Pablo Tobón Uribe, Medellín, COL; 4 Pathology, Hospital Pablo Tobón Uribe, Medellin, COL

**Keywords:** body mri, child and adolescent, renal neoplasm, sickle cell trait, renal medullary carcinoma

## Abstract

Renal medullary carcinoma (RMC) is an aggressive and rare malignancy that usually presents in adolescents and young adults with sickle cell disease. Herein, we describe a case of a white male with an unknown sickle cell trait, who presented with left iliac fossa pain, without any other finding that suggested renal neoplasia.

Imaging findings were a renal mass of central location with caliectasis, renal hilar adenopathy, and paraaortic lymphadenopathy. Biopsy confirmed an RMC diagnosis.

RMC diagnosis requires clinical suspicion in sickle cell patients who present with pain and hematuria. Imaging shows a central mass, with an infiltrative appearance, frequently associated with calyx’s dilation and lymphadenopathy. Prognosis is poor in spite of the treatment.

## Introduction

Renal medullary carcinoma (RMC) is both a malignant and infrequent tumor of epithelial origin [1-4]. RMC represents less than 0.5% of renal carcinomas [2]. Since its description in 1995 by Davis et al [5], few cases and case series have been reported [1].

RMC usually affects older children or young adults with sickle cell trait or heterozygous sickle cell disease (hemoglobin [Hb] SC disease), in the age of 5-39 years (mean age 14.8 years) [4,5,6]. Homozygous Hb SS disease is not associated with RMC [4]. In patients younger than 25 years, RMC has a higher prevalence in male sex (2-3:1, M:F); no sex differences have been observed in patients older than 25 years [5-7]. ﻿

RMC arises from renal papillae and caliceal epithelium, where drepanocytosis is higher and epithelial cell proliferation is generated. Satellite lesions are usually found in the renal cortex and soft perirenal tissues. Right kidney affection is more common [1,5-9].

Pathology shows a solid tumor, with cells in a desmoplastic stroma, acute and chronic inflammatory infiltrate, necrosis, hemorrhagic foci, and drepanocytes (sickle cells). Calcifications are rare [4,6,8,10]. RMC usually carries a loss of suppressor gene SMARCB1/INI1, and high P53 expression and KI67 index. High molecular weight keratin stains are variable [2,11]. 

The objective of this article is to describe the imaging findings in CMR based on an unusual case with an atypical clinical presentation.

## Case presentation

A 12-year-old male patient, with no personal medical history, presented to the ED due to 20 days of left iliac fossa pain, which increased over time. A review of systems was negative for fever, weight loss, hematuria, or any other urinary symptom. Physical examination found pain elicited by palpation in the left flank and positive fist percussion on the same side.

**Figure 1:**
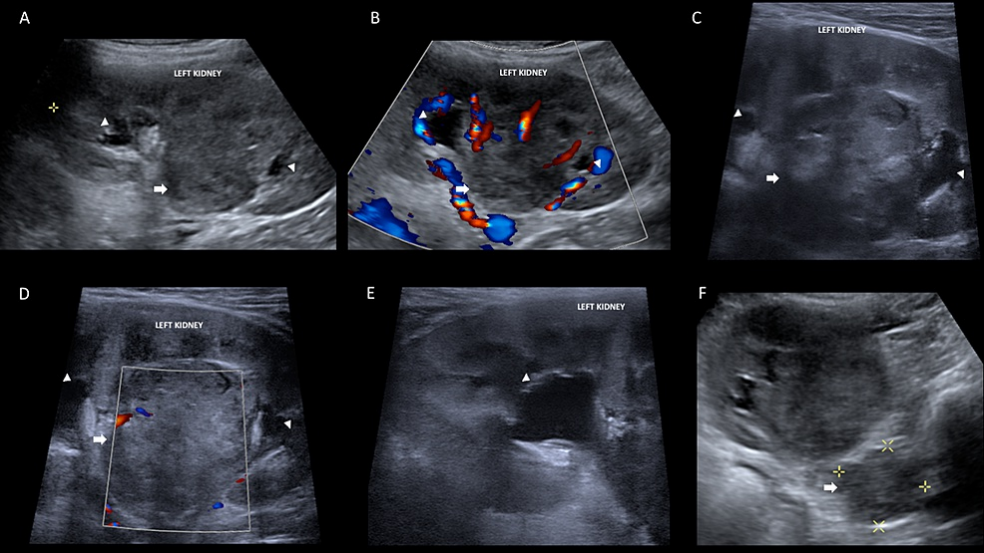
Abdominal ultrasound. Abdomen ultrasound with low-frequency convex transducer (A and F) and high-frequency linear transducer (C and E). Solid renal lesion of central location (arrow in A, B, and C), generating focal caliectasis in both superior and inferior pole (arrowhead in A, B, C, D, and E). It was hypovascular in color Doppler mode (B and D). Additionally, retroperitoneal lymphadenopathies were observed in paraaortic and renal hilum locations (arrow in F).

**Figure 2:**
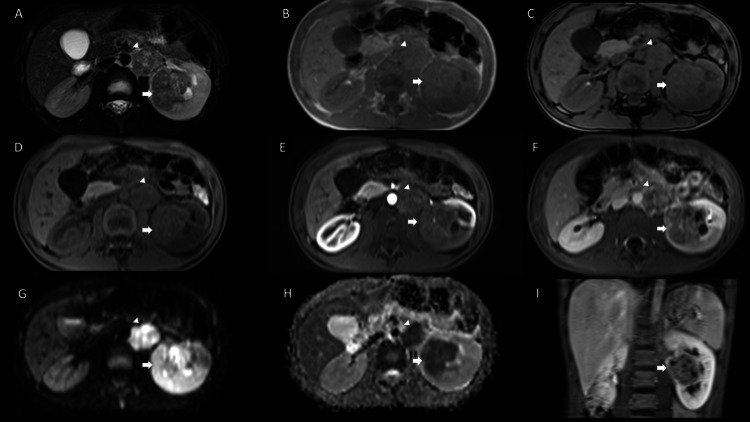
Contrast-enhanced MRI of the abdomen and pelvis. MRI on axial T2-weighted (A), in-phase (B), and out-of-phase T1 sequence (C). T1-weighted imaging with fat saturation (D), post-contrast T1-weighted with fat saturation on arterial phase (E), late phase (F), and coronal image on late phase (I). B800 diffusion sequence (G) and its apparent diffusion coefficient (ADC) map (H). Solid infiltrative lesion centrally located in left kidney, hypointense on T2W (arrow in A), with no out-of-phase signal drop in T1-weighted imaging that indicates any fat component (arrows in C and D); the intermediate signal on T1-weighted imaging (arrow in D); and hypovascular on contrast-enhanced sequences (arrows in E, F, and I). Additionally, retroperitoneal lymphadenopathies are confirmed (arrowhead in A, B, C, D, E, F, G, and H).

**Figure 3:**
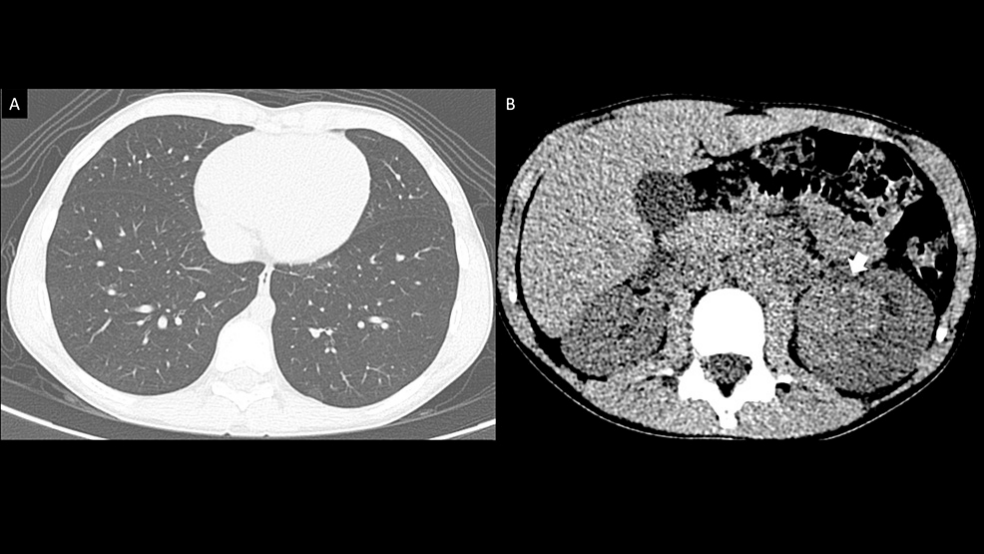
Non-contrast chest CT. Non-contrast chest CT in lung window (A) and soft tissue window (B). Lung parenchyma with no metastasis (A). Hyperdense left renal lesion (arrow in B).

**Figure 4:**
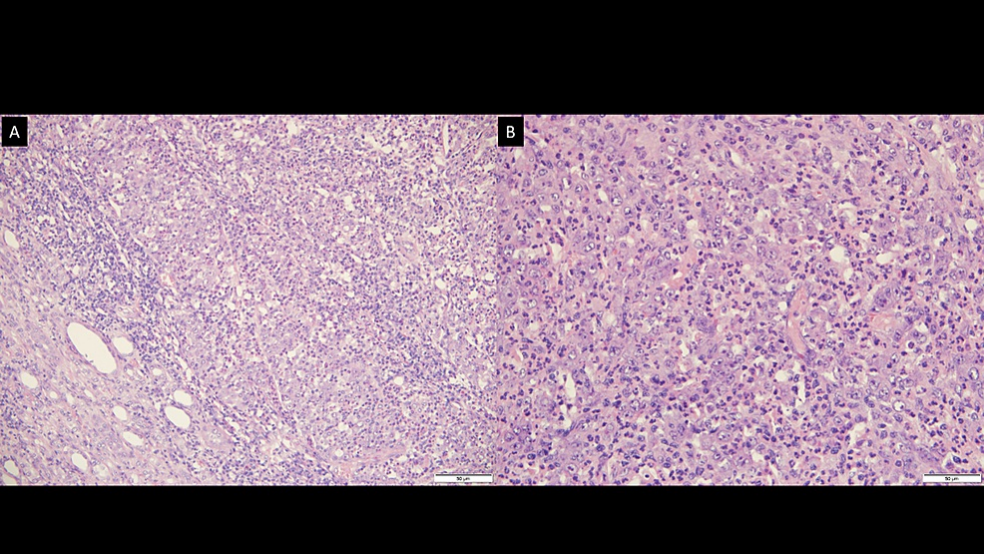
Hematoxylin and eosin (H&E) staining. Tumor with extensively atypical cells, with clear, vesiculated nuclei; prominent nucleoli, and eosinophilic cytoplasm. (A) Extensive necrosis and inflammatory component with lymphocytes, polymorphonuclear leukocytes, and macrophages. (B)

**Figure 5:**
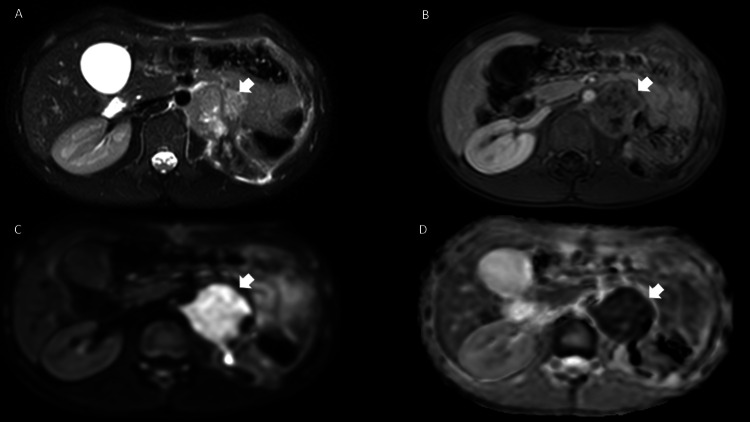
Contrast-enhanced MRI of the abdomen. Simple and contrast magnetic resonance on axial T2 sequences (A), post-contrast T1 with fat saturation (B), B800 diffusion sequence (C), and its ADC map (D). Changes due to left nephrectomy, with the persistence of retroperitoneal lymphadenopathies. (arrows in A, B, C and D).

Abdominal ultrasound and contrast abdominal MRI showed a left renal mass, retroperitoneal lymphadenopathies in renal hilum and paraaortic location, with no evidence of metastasis (Figures 1 and 2). CT of the chest ruled out pulmonary metastasis (Figure 3). The patient underwent nephrectomy and lymph node resection; total lymph node resection was not possible. Frozen section biopsy of the renal mass and lymph nodes suggested malignant neoplasia, not suggestive of lymphoma; RMC diagnosis was subsequently confirmed, with an absence of INI1 expression, and positive for EMA, AE1/AE1, and vimentin (Figure 4). Due to the strong relationship between RMC and sickle cell disease, Hb electrophoresis was performed, confirming a Hb SC disease. Platinum-based chemotherapy was initiated with no radiotherapy. The patient is alive four months after the diagnosis, with persistent retroperitoneal lymph nodes in the last imaging follow-up with MRI (Figure 5).

## Discussion

Clinical presentation includes hematuria, flank pain, weight loss, and even respiratory distress. Infrequent symptoms are palpable mass, cough, hypertension, scrotal pain, and fever [1,2,6,8].

Imaging identifies a centrally located mass, with rapid growth and infiltrative, ill-defined margins, that can extend to the renal cortex and sinus. Even though there is a renal expansion of the mass, a renal configuration is conserved. Ultrasound shows a solid mass with caliectasis but no pyelectasis, with no vascularization in color flow Doppler, and minimum peritumoral vascularization in the power Doppler mode. It is worth remarking that some tumors may not be detected through ultrasound [1,2,6,8,10]. Angiography shows the hypovascular nature of the tumor [3,6]. RMC is usually heterogeneous on CT and MRI due to necrosis areas, and lower enhancement than the adjacent renal parenchyma is typical [6,10]. The tumor may extend onto perirenal fat tissue, and it is associated with distant metastasis. Lymphadenopathy and vascular invasion are common [3,6]. MRI is superior to CT to detect both hemorrhage and hepatic metastasis [8].

Differential diagnosis includes multiple diseases. Renal lymphoma usually has an infiltrative pattern, with multifocal and bilateral involvement, associated with diffuse lymphadenopathy and multiple organ involvement. Rhabdoid tumor and mesoblastic nephroma must be distinguished from RMC, as these tumors usually present in younger patients. Even though it is infrequent in adolescents, Wilms' tumor must be taken into account in the differential diagnosis. Transitional cell carcinoma and renal cell carcinoma may look like RMC, but a loss of the SMARCB1 gene, and the association with sickle cell trait, suggest RMC. Finally, RMC may simulate pyelonephritis; therefore, history and physical examination are essential for proper diagnosis [1,2,6,10]. Age and sickle cell trait history may help to perform the correct diagnosis of RMC [6,10].

When a diagnosis is performed, the advanced disease is usually found [1,10], with hepatic, pulmonary, and lymphatic metastasis. Prognosis is poor, and survival after surgical intervention is of 4-5 months in cases with metastasis [1,2,6], with an overall mortality rate of 95% [1,2].

## Conclusions

RMC is an infrequent tumor of aggressive behavior, with a predilection for young patients with sickle cell trait or heterozygous sickle cell disease. Our case shows an unusual presentation in a white adolescent with unknown Hb SC disease. Imaging findings consist of a centrally located mass invading the renal sinus with peripheral caliectasis, but renal morphology is preserved. The prognosis of this disease is usually poor, despite treatment.
